# Supplementary material for: An Updated PAH Mutational Spectrum of Phenylketonuria in Mexican Patients Attending a Single Center: Biochemical, Clinical-Genotyping Correlations
Source: Genes (Basel). 2021 Oct 23;12(11):1676. doi: 10.3390/genes12111676 (PMC8620669; doi:10.3390/genes12111676)
Supplement: Supplementary file 1 [file genes-12-01676-s001.zip › genes-1418497-SI.pdf]

Supplementary **Table S1**. PCR oligonucleotides and sequencing conditions for *PAH* gene.

Name of primer	Sequence (5'-3')*	Size of amplicon (bp)**
**M13R-pUC-PAH-EX1F**	CAGGAAACAGCTATGACGCGAGGTTAAAACCTTCAGCCC	300
**M13F-PAH-EX1R**	GTAAAACGACGGCCAGTTGACTTCCTGGATATTCTCATCAGC	
**M13R-pUC-PAH-EX2F**	CAGGAAACAGCTATGACTGTCCATGGAGGTTTAACAGGAAT	303
**M13F-PAH-EX2R**	GTAAAACGACGGCCAGTTGAGCTCAAATTCAAATCTGCCTG	
**M13F-PAH-EX3F**	GTAAAACGACGGCCAGTGAACTAACTGCCCCACCTCC	614
**M13R-pUC-PAH-EX3R**	CAGGAAACAGCTATGACGGTCCCCAACCTATGTCCAC	
**M13F-PAH-EX4F**	GTAAAACGACGGCCAGTTTTGGGGGTTATCTGGAAGCC	367
**M13R-pUC-PAH-EX4R**	CAGGAAACAGCTATGACTTTCCCAGCCCTCGTGTAAAT	
**M13F-PAH-EX5F**	GTAAAACGACGGCCAGTAGGAGAGCTAAGTTTAACCGAGAC	417
**M13R-pUC-PAH-EX5R**	CAGGAAACAGCTATGACCATGTGTTATTAAAGGGAGGGTTT	
**M13R-pUC-PAH-EX6F**	CAGGAAACAGCTATGACATTCACTGTAGCAAGTGATGGC	515
**M13F-PAH-EX6R**	GTAAAACGACGGCCAGTGGATAAACACAGTAGGGGCTG	
**M13R-pUC-PAH-EX7F**	CAGGAAACAGCTATGACATGTCCCTGGGCAGTTATGT	423
**M13F-PAH-EX7R**	GTAAAACGACGGCCAGTGGTCTATCTACTTGGATGGAGCAA	
**M13F-PAH-EX8F**	GTAAAACGACGGCCAGTTCCCAACCTCTGCATATCACTT	412
**M13R-pUC-PAH-EX8R**	CAGGAAACAGCTATGACCCCATGCTTGATCTCCGAAA	
**M13F-PAH-EX9F**	GTAAAACGACGGCCAGTTCTGAAGGCATCTGGCCACC	260
**M13R-pUC-PAH-EX9R**	CAGGAAACAGCTATGACAGATAACCTGGCTTCCAGGGG	
**M13R-pUC-PAH-EX10F**	CAGGAAACAGCTATGACACTCACATGCCAATCCCCTC	380
**M13F-PAH-EX10R**	GTAAAACGACGGCCAGTGGAGAATGAGTTCCCAGGTTG	
**M13R-pUC-PAH-EX11F**	CAGGAAACAGCTATGACAGCATTTGGGCTGTGATGTAGA	508
**M13F-PAH-EX11R**	GTAAAACGACGGCCAGTTGTCTTGACTTGGTGGTTGC	
**M13F-PAH-EX12F**	GTAAAACGACGGCCAGTGCTTCCCAAGAAGACTCATTAAAA C	432
**M13R-pUC-PAH-EX12R**	CAGGAAACAGCTATGACCATGGCTTACATGGAGGTGCTT	
**M13R-pUC-PAH-EX13F**	CAGGAAACAGCTATGACAGCTCCTTGAAAGTTGGGGT	358
**M13F-PAH-EX13R**	GTAAAACGACGGCCAGTTGTACTCATATCCTGTCATTTCAGA	

*Universal sequencing primers are highlighted (M13F and M13R-pUC).
**Size without universal sequence.
